# ICM Implantation and Remote Follow‐Up Management by Trained Nurses in Italian Hospitals: Current Practice and Nurse Feedback

**DOI:** 10.1111/jce.16582

**Published:** 2025-01-29

**Authors:** Rosario Cervellione, Simona Fetche, Marzia Simoncelli, Paola Frasnelli, Maurizio Vargiu, Cinzia Messina, Emanuele Contu, Anna Bertazzo, Eleonora Baccolo, Marco Carconi, Francesco Cropanese, Cristina Spina, Donato Montanaro, Annalisa Mercurio, Giuliana Facchetti, Massimo Moltrasio, Stella Baccillieri, Giosué Mascioli, Massimiliano Maines

**Affiliations:** ^1^ Centro Cardiologico Monzino IRCCS Milano Italy; ^2^ Azienda Ospedaliero Universitaria Integrata di Verona Verona Italy; ^3^ Osp. S. Maria del Carmine Italy; ^4^ Osp. Centrale di Bolzano—AS Alto Adige Bolzano Italy; ^5^ Osp. Santa Maria Goretti Latina Italy; ^6^ Osp. Giovanni Paolo II ‐ ASP Ragusa Ragusa Italy; ^7^ Azienda Ospedaliera Brotzu Cagliari Italy; ^8^ Osp. San Bassiano ‐ AULSS7 Bassano del Grappa Italy; ^9^ Osp. di Desenzano del Garda ‐ ASST del Garda Desenzano del Garda Italy; ^10^ Osp. Civile di Gorizia ‐ ASU Giuliano Isontina Gorizia Italy; ^11^ Osp. San Giovanni di Dio ‐ ASP Crotone Crotone Italy

**Keywords:** ICM implantation, implantable cardiac monitor, nurse, nurse satisfaction, standard operating procedures (SOP), telemedicine

## Abstract

**Aims:**

This project aimed to evaluate current practices of trained nurses performing implantable cardiac monitor (ICM) implantations and remote follow‐ups in Italy, assessing hospital protocols and nurses' perceptions.

**Methods:**

An anonymous survey was conducted among 163 trained nurses across 75 Italian hospitals, focusing on their ICM implantation and remote monitoring practices. Data collected included hospital characteristics and protocols, barriers to implementation, and nurses' feedback on their experiences.

**Results:**

Of the 112 respondents (69% response rate), 60% reported that nurses in their hospitals are authorized to perform ICM implantations, and 70% said that they can manage all remote monitoring tasks. Thirty‐three (29%) nurses manage all aspects of ICM patient care, including implantation, programming, enrollment in remote monitoring, training, data review, and follow‐up. Fifty‐five percent of nurses perform a part of ICM implants outside the EP/Cath lab, and for 31%, this is the primary location. 84% of implanter nurses achieved autonomy after < 10 supervised implants. More than 90% of implanter nurses consider ICM implantation rewarding and 96% find it safe and easy with the provided kit. However, only 33% and 17% of nurses had written protocols at their hospital, to guide ICM implantation and remote monitoring, respectively.

**Conclusions:**

Nurse‐led ICM implantation and remote follow‐up are becoming established practices in Italy, with many nurses operating independently. Despite this progress, the absence of standard operating procedures limits the widespread adoption of these practices. Clear national and international protocols are essential to enhance nurse training, ensure safe practices, and ultimately improve patient care in ICM management.

## Graphical abstract

**Figure:**
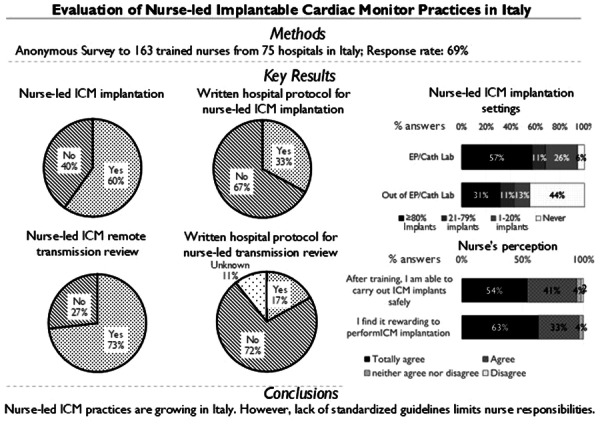
Key results of the survey about nurse‐led implantable cardiac monitor management practices in Italy.

## 1 Introduction

Implantable loop recorders (ILRs) are recommended by international guidelines [1, 2] for diagnosing unexplained syncope or falls, palpitations, cryptogenic stroke and monitoring atrial fibrillation recurrence following AF ablation, as they offer a higher diagnostic yield than intermittent monitoring tools [3, 4]. The latest ILR generation has been miniaturized and redefined as insertable cardiac monitors (ICM), allowing a minimally invasive insertion under the skin using a dedicated tool [5, 6, 7, 8].

In recent years, nursing skills in cardiology have evolved beyond historical responsibilities, especially in telecardiology and interventional electrophysiology, with a special focus on cardiac pacing and related monitoring services. Reports have shown that trained arrhythmia nurses can safely implant ICMs autonomously [9, 10, 11, 12, 13, 14, 15, 16, 17]. Moreover, nurse‐led ICM implantations have been as safe and effective as EP‐led ones [8, 9, 10, 11, 12, 13, 17] while also improving hospital efficiency [9, 11, 12] and reducing costs, particularly when procedures move from the electrophysiology or catheterization laboratory (EP/Cath lab) to out‐patient settings [11, 12].

In response to the growing number of UK hospitals allowing non‐medical staff to perform ICM implantation, the British Heart Rhythm Society (BHRS) released evidence‐based guidance in 2018, updated in 2020 [18], providing minimum standards for safe ICM implantation and management by nurses or cardiac physiologists. Following this success, in Italy, ICM manufacturers conceived training centers in selected hospitals, for nurses to receive theoretical and practical training by electrophysiologists or expert nurses on ICM indication, implantation, and remote monitoring. However, no guidelines from the Italian Arrhythmology and Cardiac Pacing Association have been published, leading some hospitals to create internal standard operating procedures (SOP) to authorize trained nurses to implant ICMs and/or manage remote follow‐ups, while others allow this only under electrophysiologist responsibility.

Our project aimed to assess the rate of trained nurses currently performing ICM implantation and remote follow‐up in Italian hospitals, and to characterize hospital protocols and current practice. Additionally, we sought to investigate the nurse's perception and gather feedback on aspects of current practice, including the ease and safety of ICM implantation, confidence and satisfaction with their new responsibilities, implant tools, ICM programmer/remote monitoring systems, and training materials.

## 2 Methods

This project was based on a survey sent to nurses who have received a training course about ICM implantation and remote follow‐up in Italy.

The survey was fully anonymous, and no sensitive data were collected or managed. Participation of survey recipients was voluntary. The project was conducted in accordance with local laws and regulations.

### 2.1 Project Phases

In October 2023, an Expert Nurse Focus Group (ENFG) of 15 expert nurses in cardiac pacing, each with over 2 years of experience in ICM implantation and/or remote monitoring, met to share best practices. Four electrophysiologists also attended to support the group. During the meeting, the ENFG designed a survey to gather current practice and feedback from nurses who had previously trained in ICM implantation and management.

The survey was designed to collect data on:

− the percentage of trained nurses allowed to perform ICM implantation and/or remote follow‐ups at their hospital, barriers to implementation, existing hospital SOP, and current practices in Italian hospitals,
− nurses' perceptions of the ease and safety of ICM implantation, satisfaction and confidence in this new role, and feedback on tools and trainings.

A draft of the questionnaire was created during the meeting and shared by email with all participants to refine and finalize all the questions. An electronic form was developed using Fillout.com service and invitations to participate were sent three times between November 2023 and March 2024 to the e‐mail addresses of all nurses who had previously attended an ICM training course. The service saves the survey responses only if all questions have been answered. Data collection closed in April 2024 and responses were processed.

### 2.2 Survey Structure

The survey included 65 multiple choice and ranking questions covering (see Appendix A): (1) Hospital information and available standard operating procedures (SOP) for nurse ICM implantation; (2) Nurse experience; (3) Nurse current practices about ICM implant procedure, settings, and materials; (4) Nurse feedback and satisfaction with ICM implantation; (5) ICM remote monitoring, available SOP and nurse involvement; and (6) Nurse feedback about patient enrollment in the remote monitoring system, training materials for nurses, and patients and ICM data transmission review.

### 2.3 Statistics

The results of the survey were presented with descriptive statistics. Categorical variables were expressed as counts and percentages. The chi‐square test for categorical variables was used to assess the significance of differences between groups. Statistical test deemed statistically significant if *p* < 0.05. All analyses were performed using SAS 9.4 version software (SAS Institute Inc., Cary, NC, USA).

## 3 Results

The survey was sent by e‐mail to 163 nurses (from 75 Italian hospitals) who completed training on ICM implantation and remote follow‐up. By the end of data collection, 112 nurses responded, representing a 69% response rate. Of these, 86 (77%) had over 3 years of experience in the cardiac pacing area, 17 (15%) between 1 and 3 years, and 9 (8%) less than 1 year.

### 3.1 ICM Implantation by Trained Nurses

#### 3.1.1 Hospital Information and Available SOP on Nurse‐Led ICM Implantation

Table 1 reports characteristics of hospitals where survey respondents work and available SOP for nurse‐led ICM implantation. Over half of responding nurses (51%) are from southern Italy or the islands, 41% from the north, and 8% from the center. Public hospitals represented 89%, with 42% categorized as high‐volume and 49% as medium‐volume ICM implant centers. Seventy‐nine nurses (70%) have access to multiple ICM technologies.

**Table 1:** Characteristics and protocols of hospitals where interviewed nurses work, regarding ICM implantation.

Item	*n*	%
Geographical area (*N* = 112)		
Nord	46	41
Center	9	8
South and Island	57	51
Type of hospital where nurse works (*N* = 112)		
Public	100	89
University public	10	9
Affiliated private	2	2
Number of ICM implants/year at hospital (*N* = 112)		
10–20	7	6
20–50	55	49
> 50	47	42
Unknown	3	3
ICM brand implanted at hospital (*N* = 112)		
Medtronic	109	97
Biotronik	73	65
Abbott	43	38
Boston Scientific	8	7
ICM implanted by nurses at hospital (*N* = 112)		
Yes	67	60
No	45	40
Additional operators involved in ICM implants performed by nurses (*N* = 67) (excluded supervisor during training implants)		
Implanter nurse is alone	18	27
1 Allied healthcare professional (nurse/healthcare assistants or cardiology technicians)	26	39
1 Physician	7	10
1 Physician + 1 allied healthcare professional	13	19
2 Allied healthcare professionals	3	5
ICM explanted by nurses at hospital (*N* = 112)		
Yes	34	30
No	78	70
Availability of SOP on nurse‐led ICM implantation at the hospital where nurses can implant (*N* = 67)		
Yes	22	33
No	45	67
Definition of minimum number of ICM implantation procedures by nurse before being considered autonomous (*N* = 67)		
Yes	24	36
No, the supervisor decides when nurse autonomy is achieved	43	64
Defined minimum number of implants by nurse with a supervisor before autonomy (*N* = 24)		
3	2	8
5	8	34
10	2	8
≥ 20	12	50
Written documentation of ICM performed by nurses under supervision + date of achieved autonomy at the hospital (*N* = 67)		
Yes	25	37
No	42	63
Need for periodic reassessment of nurses to continue implanting ICMs (*N* = 67)		
Yes	15	22
No	52	78
Written tracking system of peri‐procedural complications at the hospital (*N* = 112)		
Yes	56	50
No	56	50
ICM complication monitoring methods (*N* = 112)		
Education of patient about possible complications and ask him to report them by phone if they occur	63	56
Schedule a visit after implantation to check wound	52	46
Patient interview about ICM complication occurrence during in‐office or telephone follow‐ups	30	27
Nurse manages ICM programming (according to physician indications) at his hospital (*N* = 112)		
Yes, alone	77	69
Yes, together with a cardiology technician	8	7
No, a cardiology technician manages it	11	10
No, a physician manages it	16	14

Abbreviation: SOP = standard operating procedures.

**Figure 1:**
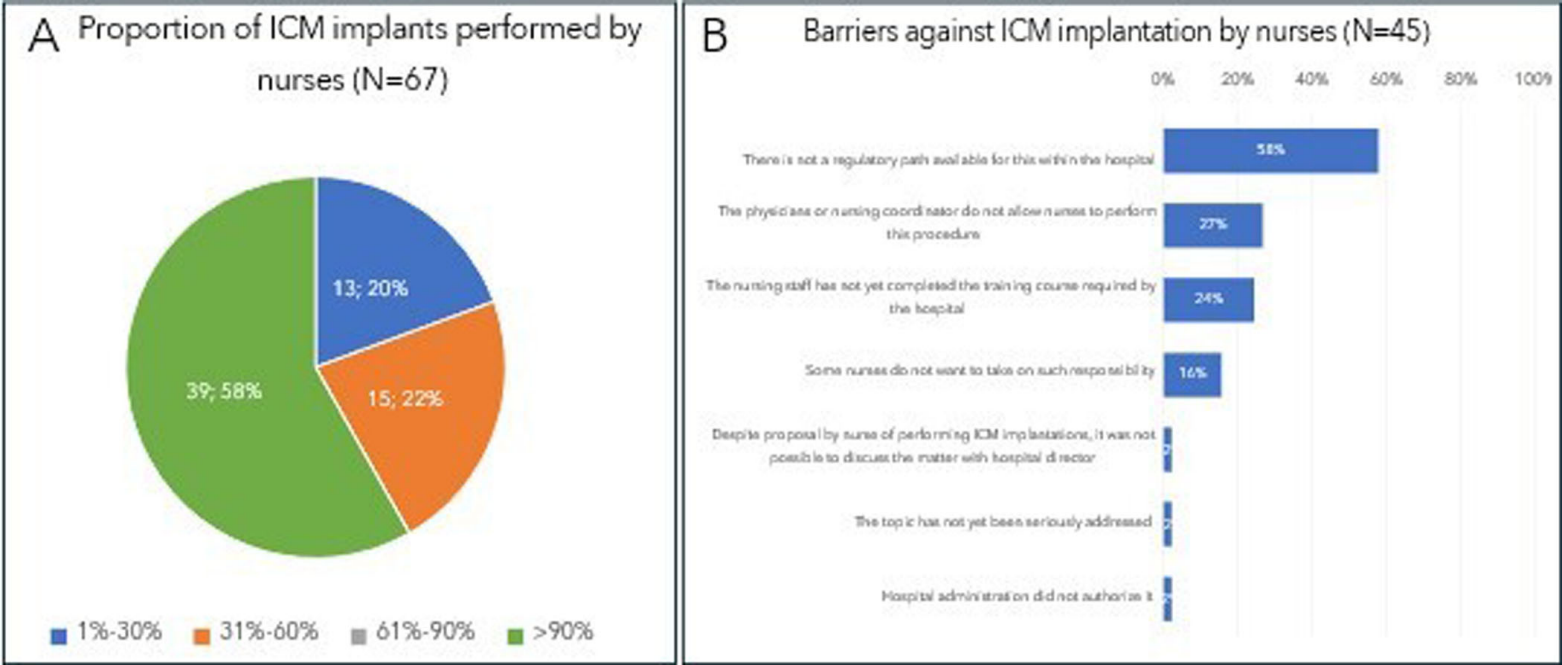
Proportion of ICM implants performed by trained nurses in the hospitals where nurses implant ICMs (A) and barriers against ICM implantation by trained nurses in the hospitals where nurses are not allowed to implant ICMs (B).

Sixty‐seven nurses (60%) indicated that trained nurses currently implant ICMs at their hospital, while 34 (30%) also explant them. Figure 1 illustrates the proportion of ICM implants performed by trained nurses in the hospitals where nurses can implant ICMs (panel A) and barriers faced in hospitals where they are not allowed to implant. In hospitals where nurses implant ICMs, 29% require physician attendance.

Among hospitals where nurses implant ICMs, 22 (33%) survey respondents reported the availability of SOP for this purpose. Additionally, 36% (64% in sites with SOP and 22% in sites without SOP) reported a minimum number of supervised implants (by an electrophysiologist or highly experienced nurse in ICM implantation) required for autonomy, with 37% (77% in sites with SOP and 18% in sites without SOP) reporting the availability of written documentation of these procedures and date of achieved autonomy. The minimum number of required supervised implants ranges from 3 (8%) or 5 (34%) to ≥ 20 implants (50%). Fifteen nurses (22%) indicated that a periodic reassessment by a supervisor is necessary to continue implanting ICMs.

A tracking system of peri‐procedural complications, including those related to ICM implantation, is available in the hospital of 50% of the total survey respondents. Complication monitoring strategies include educating patients about possible complications and reporting by phone (56%), scheduling follow‐up visits (46%) for wound check from 7 (34, 30%) to > 15 days (4%) days after implantation and patient interview during in‐office or telephone follow‐ups (27%).

#### 3.1.2 Information About ICM Implanter Nurses and Current Practices

Out of 112 surveyed nurses, 54 (50%) perform ICM implantation at their hospital. Of these, 33 (29%) also manage all other aspects of ICM patient care, including ICM programming, enrollment in remote monitoring, training, data review, and follow‐up. Details on ICM implanter nurses and their practices are reported in Table 2. Most implanter nurses work at high‐volume sites (48%) and are located both in the north (46%) and in the south or islands (41%) of Italy. Eighty‐five percent have over 3 years of cardiac pacing experience, with 57% having performed more than 20 ICM implants. For 28 (52%) implanter nurses, fewer than 5 supervised implants were needed to achieve autonomy, while 9 (16%) required ≥ 10 implants. Fifty‐one (94%) nurses reported that no patients have ever refused to be implanted by a nurse.

**Table 2:** Information about ICM implanter nurses and current management of ICM implantation.

Item	*n*	%
Geographical area (*N* = 54)		
Nord	25	46
Center	7	13
South and Island	22	41
Number of ICM implants/year at hospital (*N* = 54)		
10–20	4	7
20–50	24	44
> 50	26	48
Experience in cardiac pacing area (*N* = 54)		
< 1 year	2	4
1–3 years	6	11
> 3 years	46	85
Reason to be trained and involved as ICM implanter (*N* = 54)		
It was an imposition by hospital	1	2
Other nurses do it and I do not want to be outdone	0	0
I consider it a professional growth	49	91
I do it, but I would like to be recognized	24	44
Number of ICM implants with supervisor before autonomy (*N* = 54)		
< 5	28	52
5–10	17	32
10–20	4	7
> 20	5	9
Any implantation outside the EP/Cath Lab (*N* = 54)		
Yes	30	55
No	24	45
Physician location during ICM implantation (*N* = 54)		
Same room	13	24
Nearby room	17	31
On the same floor	21	39
In another floor	3	6
Implanter nurse clothing during ICM implantation (*N* = 54)		
Cap	48	89
Surgical mask	54	100
Aseptic gown	50	93
A pair of sterile gloves	47	87
2 Pairs of sterile gloves	7	13
Protective glasses	15	28
EKG patient monitoring during ICM implantation (*N* = 54)		
Yes	38	73
No	16	27
Patient venous access creation before ICM implantation (*N* = 54)		
Yes	36	69
No	18	31
Temporary stop OAC therapy (*N* = 54)		
No	31	57
Yes, 12 h before ICM implantation	4	7
Yes, 24 h before ICM implantation	16	30
Yes, 48 h before ICM implantation	3	6
Peri‐procedural antibiotic therapy (*N* = 54)		
No	27	50
Yes, in all patients	19	35
Yes, in patients with high infection risk	8	15
Local anesthetic drugs (*N* = 54)		
Mepivacaine	28	52
Lidocaine	24	44
Ropivacaine	4	7
Sodium bicarbonate	1	2
Use of the injection kit provided by the manufacturer (*N* = 54)		
Only the injection kit	50	93
Injection kit + additional scalpel	4	7
Adherence to ICM implant position and orientation in the patient chest manufacturer guidelines (*N* = 54)		
Yes, if patient's anatomy allows it	29	54
Yes, if patient anatomy allows it, but with exception of women with breast implants	18	33
No, pre‐implant ECG mapping is performed	4	7
No, decision per implanter discretion	3	6
Wound closure methods (*N* = 54)		
Steri‐Strips	30	56
Absorbable stitches	25	46
Nonabsorbable stitches	18	33
Skin glue	5	9
Wound dressings (*N* = 54)		
Medicated transdermal patches	45	83
Medicated transdermal patches + application of ice and weight	1	2
Advanced dressings (e.g., silver‐impregnated dressings)	4	7
Compressive dressings	8	15
Common gauze and plaster	1	2

**Figure 2:**
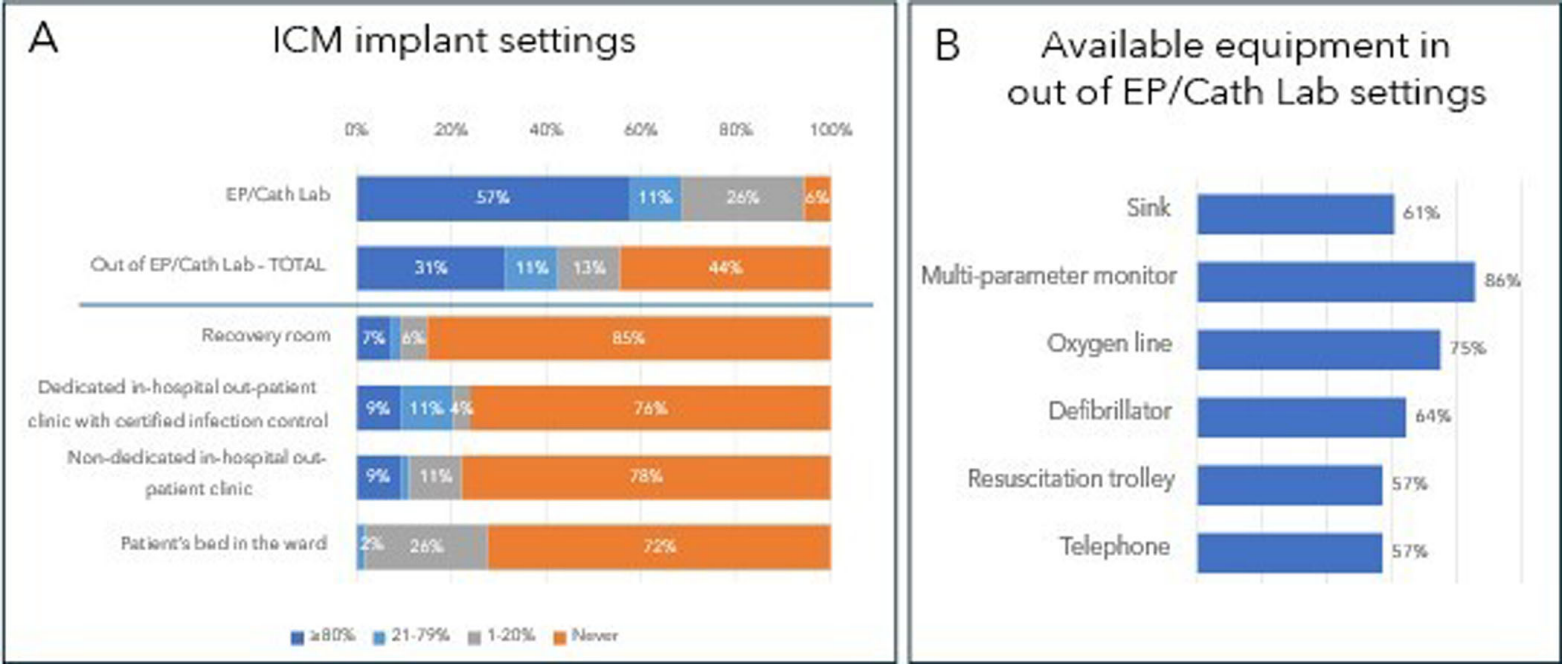
Implant settings where nurses perform ICM implantation (A) and available equipment in non‐standard setting (B).

Regarding ICM implant location (Figure 2A), 29 (55%) nurses use settings outside of the EP/Cath lab for all or part of ICM implants and 17 (31%) use this as the primary location (> 80% of implants performed there). On the other hand, 57% still predominantly use the EP lab/Cath lab. Figure 2 also reports the equipment available in out of EP/Cath lab settings (Figure 2B): when not present in the implanting room, resuscitation equipment is always nearby. During nurse‐led ICM implantation, a physician is in the same room for only a quarter of cases (24%), while he is often in a nearby room (33%) or on the same floor (36%).

During ICM implantation, it is nurse's common practice (73%) to monitor patient's electrocardiogram (EKG) with a multiparameter monitor and to establish venous access beforehand (69%). These practices are more frequent for nurses who only implant in the EP/Cath lab (100% vs. 53% for EKG monitoring; *p* < 0.01% and 88% vs. 53% for venous access; *p* < 0.01). For patients on anticoagulants, 57% of nurses do not stop the therapy, while others typically stop it 24 h (30%), 12 h (7%), or 48 h (6%) before the procedure. Fifty percent of implanter nurses do not administer peri‐procedural antibiotics for ICM implants, while 35% give them to all patients and 15% only to those at high infection risk. All nurses use the manufacturer's implantation kit, though four (8%) use an additional scalpel; most (87%) follow manufacturer guidelines for ICM position and orientation in the patient's chest, but for 33% of them, an exception should be considered for women with breast implants. Preferred wound closure methods are Steri‐Strips (56%), absorbable (46%), and nonabsorbable (33%) stitches, and the most common dressing is medicated transdermal patches (85%) (Table 2).

After implantation, nurses perform ICM programming, following physician's indications, in the hospitals of 76% of surveyed ones, while 10% have a cardiology technician doing it, and 14% have a physician handle it (Table 1).

#### 3.1.3 Nurse Feedback and Satisfaction on ICM Implantation

**Figure 3:**
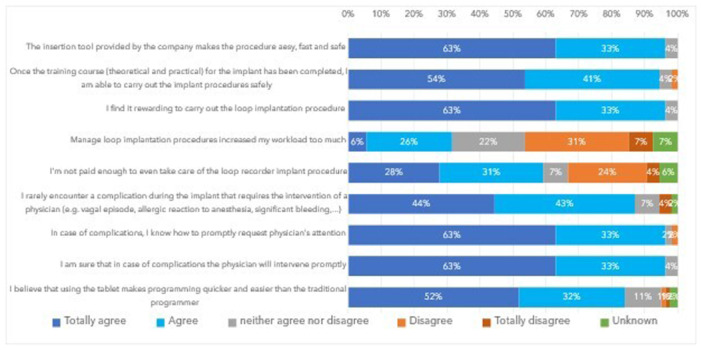
Nurse feedback and satisfaction on ICM implantation.

Among 54 nurses who perform ICM implantations, 49 (91%) chose this role for professional growth, while 24 (44%) seek recognition for this activity, and 1 nurse (2%) had this decision imposed by the hospital (Table 2). For 30 (56%) nurses, professional growth was the sole reason for their choice. As shown in Figure 3, nurse feedback about ease and safety of ICM implantation procedure, confidence in performing it and in managing potential complications, and about the programmer tablet was positive for the majority. Most nurses consider ICM implantation a rewarding activity, although the increased workload and the lack of additional compensation are concerns for 31% and 59% of them, respectively.

### 3.2 ICM Remote Monitoring by Trained Nurses

#### 3.2.1 Hospital Information and Available SOP on Nurse‐Led ICM Remote Monitoring

Table 3 displays hospital characteristics and available SOP for ICM remote monitoring by trained nurses. Over 70% of surveyed nurses reported that their hospital allows nurses to handle patient enrollment in the remote monitoring website (74%), provide them training on remote monitoring and recording activation during symptoms (75%) and/or conduct initial review of ICM data transmissions (73%). In contrast, these tasks are assigned to physicians in 14%, 13%, and 11% of cases, respectively. A cardiology technician manages remote monitoring in most other instances, while in only 2% of cases, patient training is handled by an external service provided by ICM manufacturer. In the hospital of 69 (62%) interviewed nurses, no SOP exists to indicate which ICM data transmissions should be notified to physicians after the first nurse review. However, it is the current practice of 41 (37%) nurses to notify all transmissions with new or questionable arrhythmic episodes, of 24 (21%) to notify arrhythmic episodes only when no clinical action has been taken yet (e.g., anticoagulant initiation), of 15 (13%) to notify all the transmission with episodes regardless of type or action. Additionally, 58 (52%) nurses provide the recording tool for symptoms to over 75% of ICM patients.

**Table 3:** Characteristics and protocols of hospitals where interviewed nurses work, related to ICM remote monitoring.

Item	*n*	%
Number of ICMs managed through remote monitoring at hospital (*N* = 112)		
< 50	31	28
50–200	51	45
200–500	23	21
> 500	7	6
% of implanted ICM provided with remote monitoring (*N* = 112)		
< 25%	7	6
25%–50%	3	3
50%–75%	5	4
75%–95%	10	9
> 95%	87	78
The nurse manages patient enrollment in remote monitoring system at the hospital (*N* = 112)		
Yes	83	74
No, a cardiology technician manages it	13	12
No, a physician manages it	16	14
Enrollment of patient in remote monitoring system at hospital (*N* = 112)		
At implant	99	88
After implant, but pre‐discharge	13	12
The nurse manages patient training about remote monitoring and symptom recording at the hospital (*N* = 112)		
Yes	84	75
No, a cardiology technician manages it	11	10
No, a physician manages it	15	13
No, an external service provided by ICM manufacturer manages it	2	2
Possibility to withdraw remote monitoring in the patient informed consent form of the hospital (*N* = 112)		
Yes	44	39
No	68	61
The nurse performs first review of remote data transmissions and notifies the physician about the relevant one, at the hospital (*N* = 112)		
Yes	82	73
No, a cardiology technician manages it	18	16
No, a physician manages it	12	11
Availability of SOP of the hospital indicating which transmissions/events need to be notified to the physician (*N* = 112)		
Yes	19	17
No, but nurse and physician agree on which transmissions/events need to be notified	69	62
No, a physician reviews ICM transmissions	12	11
Unknown	12	11
Type of transmissions to be notified to the physicians (*N* = 112)		
All transmissions containing arrhythmic episodes	15	13
Transmissions containing new and dubious arrhythmic episodes	41	37
Transmissions containing new and non‐managed arrhythmic episodes	24	21
Unknown (interviewed nurse not involved in transmission review)	32	29
ICM patients with tool (activator/App) for recordings of symptoms (*N* = 112)		
> 75%	58	52
25%–75%	25	22
< 25%	29	26

Abbreviation: SOP = standard operating procedures.

#### 3.2.2 Nurse Feedback and Satisfaction on Training Materials and Remote Monitoring

**Figure 4:**
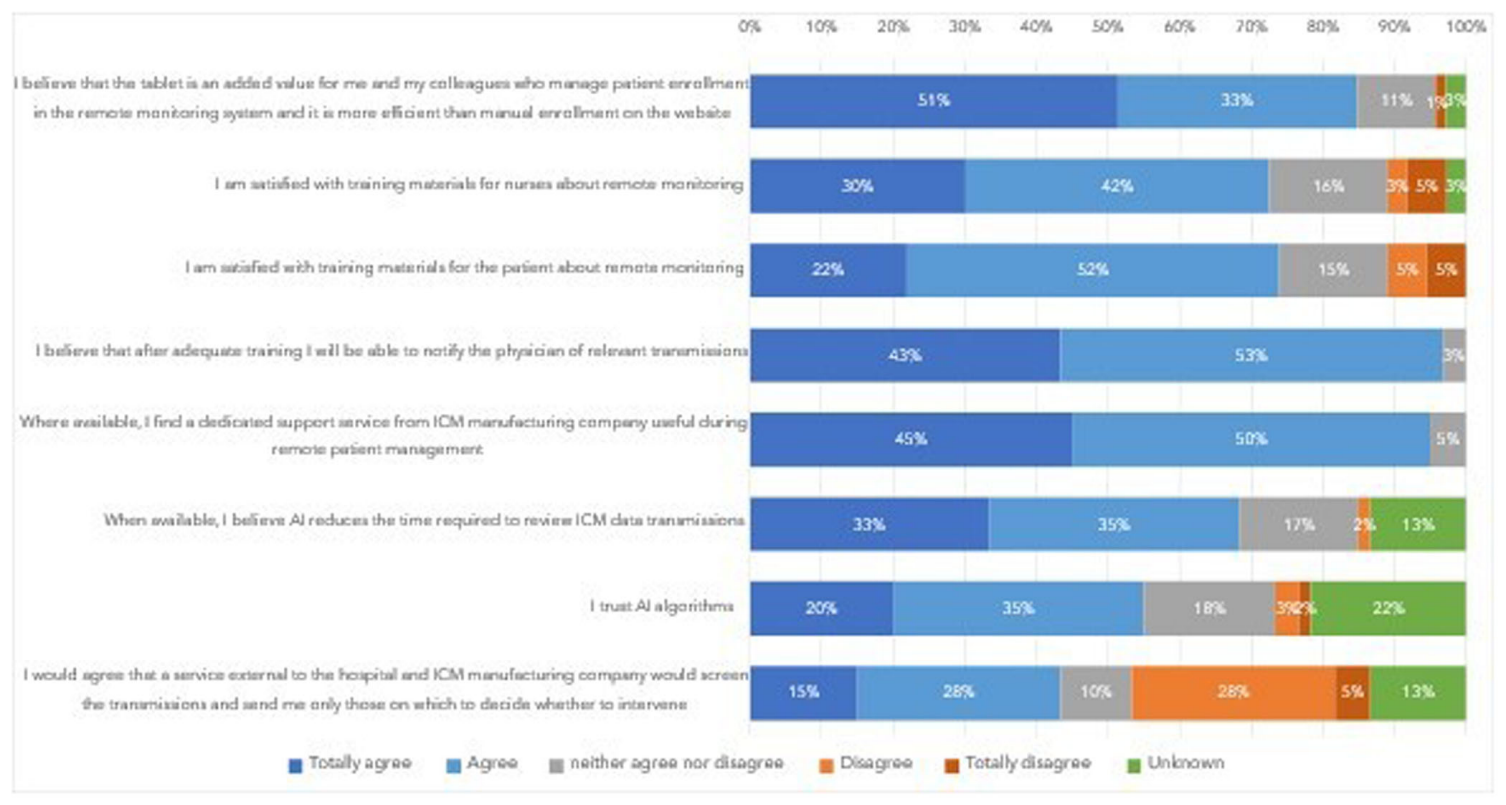
Nurse feedback and satisfaction on training materials and remote monitoring.

Among the 112 interviewed nurses, 72 (64%) personally manage patient enrollment in the remote monitoring system, 73 (65%) provide patient training on ICM functioning, remote monitoring, and activation recording, and 60 (54%) review ICM data from the remote monitoring system. Figure 4 shows their feedback and satisfaction with remote monitoring tools and training materials. Eighty‐four percent find the tablet a more efficient tool for patient enrollment compared to manual method. Almost all nurses involved in remote data review feel confident in notifying physicians of relevant transmissions after adequate training and find the support from a dedicated service provided by the ICM manufacturer helpful. Two‐thirds believe that AI can reduce transmission review time, and 55% already trust AI algorithms. The use of an external service (to the hospital and to the ICM manufacturer) to screen ICM transmissions and to notify the hospital only the actionable ones is considered acceptable by 43%.

## 4 Discussion

In recent years, about 160 experienced nurses from over 75 sites in Italy have been authorized to attend training courses for ICM implantation and remote follow‐up management. Our survey of these trained nurses shows that nurse‐led ICM implantation and remote follow‐up are currently established practices in Italy, with all interviewed nurses managing at least one task related to this process. Approximately 30% of them are accountable for the complete management of patients with ICM, involving implantation, programming, enrollment in remote monitoring systems, patient training, and data transmission review. However, many hospitals lack SOP, leaving final responsibility and level of nurse involvement in ICM implantations and remote data review at the discretion of physicians.

### 4.1 ICM Implantation by Trained Nurse

#### 4.1.1 Ease and Safety of the Procedure and Nurse Empowerment

The miniaturization of devices and the use of the dedicated implantation tool have made ICM implantation safely feasible by trained allied professionals [9, 10, 11, 12, 13, 14, 15, 16, 17]. Nurse‐led ICM implantation has been shown to improve hospital efficiency [9, 12, 13] and reduce costs, particularly in outpatient settings [12, 13]. Key findings from our survey indicate that 60% of Italian nurses trained in ICM implantation are allowed to perform the procedure at their hospital, with many performing over 90% of these procedures. Nurse implanters are well distributed across Italy. However, the lower proportion of survey respondents (8%) and implanter nurses (13%) from the center of Italy may reflect the lack of a training center in that area.

After the initial training, only 29% of nurses involve a physician during the procedure. In fact, a significant majority find the procedure easy, fast, and safe with the provided implantation kit. After adequate training, they feel prepared to safely carry out the procedure and manage potential rare complications (87% of nurses report rare occurrences) with prompt physician support, if necessary.

Nurse‐led ICM implantation results in nurse empowerment. More than 90% of implanter nurses consider ICM implantation rewarding and a form of professional growth, although the increased workload and the lack of additional compensation are concerns for some.

#### 4.1.2 Lack of SOP

Nurse unwillingness to take on ICM implant responsibility was considered a barrier hindering this program only for a low percentage of respondents. The results of our survey show that the main obstacle is the lack of SOP in hospitals. Consequently, some physicians or nurse coordinators are afraid to take on the responsibility of authorizing nurse‐led implantation, which was reported as the second most important barrier in our survey. In the UK, as more centers allowed nurses or cardiac physiologists to perform ICM implants, BHRS issued evidence‐based guidance [18] to set minimum standards for their safe ICM implantation and management. On the contrary, in Italy and the rest of Europe, despite more centers adopting this strategy recently, scientific societies have not yet released guidelines, and hospital SOP remain non‐standardized, loosely based on BHRS standards [18]. Our survey shows that, regardless of available hospital SOP, most Italian hospitals/nurses are adhering to room, equipment, staffing, implantation tools and most of the procedure requirements of BHRS standards [18]. However, they often deviate from these standards, regarding the following aspects, that should be urgently addressed by national guidelines/standards to increase the adoption of this safe and cost‐saving strategy in Italy: the minimum number of 20 implants for assessing nurse competency, nurse competency reassessment if 20 procedures/year are not performed, the need of a hospital‐certified for infection‐control implantation room, uninterrupted oral anticoagulant therapy strategy during the procedure, the use of peri‐procedural antibiotics and the possibility of ICM explant procedure by nurses.

#### 4.1.3 Number of Supervised Implants for Competency

We found that, in the practice of Italian sites, many nurses achieved autonomy after less than 10 supervised implants (52% after less than 5) and no reassessment is requested for about 80% of trained nurses, regardless of the amount of procedures/year. These results suggest, along with the infrequent occurrence of complications [9, 10, 11, 12, 13, 14, 15, 16, 17], even with only three to five supervised nurse procedures to assess competency [11, 12, 16], that shortening the practical training phase and avoiding yearly nurse reassessment could be considered by national guidelines to enable also low volume sites to implement nurse‐led implantation program and have autonomous implanter nurses more quickly.

#### 4.1.4 ICM Implant Location

Another important finding of our survey is that 55% of nurses perform ICM implants also outside the EP/Cath lab, in a location typically equipped as indicated by BHRS standards [17], and for 31% of them, this setting is the primary choice. However, a dedicated outpatient clinic with hospital certification for infection control is not always available at the hospital and even the recovery room adjacent to the EP lab, a non‐dedicated out‐patient clinic and patient's bed in the ward are also used for ICM implantation by 15%, 22%, and 28% of nurses, respectively. Future national and international guidelines should consider that multiple published experiences of ICM implantation by nurses, performed in rooms non‐certified for infection control, have reported a low complication rate (≤ 2%) [10, 11, 13, 14, 17]. Therefore, ICM implantation in non‐certified infection control rooms should be allowed, provided that all necessary equipment are available and adequate infection control strategies are applied [17]. This could reduce the percentage of procedures performed in the EP/Cath lab, which currently serves as the primary procedure location for 57% of nurses, despite its lower efficiency and higher costs [9, 12, 13, 19].

#### 4.1.5 Anticoagulation

Although BHRS guidelines [18] suggest that interrupting direct anticoagulants (DOACs) or vitamin K antagonists (VKA) before ICM implantation is generally unnecessary, 57% of Italian implanter nurses continue this practice. The primary risk for patients undergoing anticoagulation therapy during device implantation procedures is the development of a pocket hematoma, which can lead to serious consequences. This may require extended discontinuation of anticoagulation [20, 21], thereby heightening the risk of thromboembolism and infection [22, 23]. In patients on VKA anticoagulation undergoing pacemaker or defibrillator implantation, continuing VKA therapy without interruption significantly reduces pocket hematoma compared to interruption with heparin bridging, without increasing surgical or thromboembolic risks [20]. Additionally, pre‐procedural INR ≤ 2.5 in lower‐risk patients (CHA₂DS₂‐VASc ≤ 5, no mechanical heart valves, or severe mitral stenosis) and INR > 2 in higher‐risk patients significantly lowers hematoma and infection rates [24]. For patients on DOAC therapy, brief interruptions for procedures or surgery increased the risk of stroke/systemic embolism about threefold in trials comparing DOACs to VKAs [25, 26]. The BRUISE CONTROL‐2 trial showed that an uninterrupted DOAC strategy during pacemaker/defibrillator implantation is not inferior to interrupted DOACs regarding pocket hematoma and stroke incidence [27]. However, more recently, a minimal peri‐procedural DOAC interruption (no more than 24 h before and 12–24 h after the procedure) was found to be as safe as uninterrupted VKA therapy [28, 29]. During ICM implantation, the procedure is very brief [11, 12, 15] and far less traumatic than pacemaker or defibrillator implantation due to the smaller device size and simpler technique. Consequently, the risk of peri‐procedural bleeding or hematoma is very low [9, 12, 13, 15, 16, 17, 30]. Conversely, patients receiving an ICM for atrial fibrillation management or previous cryptogenic stroke often have a high thromboembolic risk. Reports of ICM implantation without anticoagulant interruption show very low rates of bleeding or hematoma [9, 16, 17, 19, 30]. Thus, it appears reasonable not to interrupt VKA (with INR on the day of surgery below the upper therapeutic range) or DOACs before ICM implantation, especially in high‐risk patients, as already currently recommended for pacemaker or defibrillator procedures [31, 32]. For low‐risk patients, minimally interrupted DOACs or VKA without heparin bridging (last dose the morning before the procedure, resumed 12–24 h later) is a safe alternative [32].

#### 4.1.6 Use of Prophylactic Antibiotics

While BHRS considers the use of prophylactic antibiotics in high‐risk infection patients [18], current practices in Italy vary, with 15% administering them in high‐risk cases and 35% doing so in general. Several studies, with the use of prophylactic antibiotics in 28%–100% of cases, have reported a very low infection risk (≤ 1.1%) [9, 11, 12, 14, 16, 19, 30]. Additionally, a non‐randomized comparison of infection rates in ICM implantation procedures, with or without peri‐procedural antibiotic administration, confirmed that most procedures are performed without antibiotics in real‐world settings, with a similarly low infection risk (< 1%) [33]. Considering the potential for reduced global antibiotic efficacy, allergic reactions, and gastrointestinal complications from unnecessary use, it is reasonable to limit antibiotics to patients at high risk of infection.

#### 4.1.7 ICM Explant Procedure by Trained Nurses

The BHRS recommends that ICMs be removed by a qualified practitioner, whether physicians, nurses, or physiologists, upon battery depletion [18]. However, only 30% of respondents to our survey reported that nurses are also authorized to perform ICM explantations at their hospitals. This may be due to limited evidence regarding the safety of ICM explant procedures performed by nurses. A retrospective single‐site study in Italy reported a 100% success rate for nurse‐led ICM explants, with no serious complications in 114 procedures performed by nurses in the EP lab with prophylactic antibiotics, compared to 88 procedures performed by electrophysiologists [34]. Two single‐site observational studies demonstrated that ICM explants or replacements can be safely performed outside of EP/Cath labs with short procedure times and no need for antibiotics, in 51 and 119 procedures, respectively, without infections or other complications [35, 36]. These findings suggest that such procedures could also be safely carried out by trained nurses. However, future multicenter comparative studies on the safety and cost‐effectiveness of nurse‐led vs. physician‐led ICM management should also include ICM explant procedures to confirm how nurses can safely manage the full ICM lifecycle.

### 4.2 ICM Remote Monitoring by Trained Nurse

For an optimal ICM diagnostic yield [37], patients should be enrolled in remote monitoring programs before discharge [38] (Class I, Level C recommendation), even if this can increase hospital workload for data review [39, 40] and require additional staff.

Our survey indicates that 72% of trained nurses work in hospitals with a significant number of remote‐monitored ICM patients, confirming the high workload that necessitates nurse involvement. Most likely for the same reason, nurses manage all phases of remote monitoring in over 70% of cases, even if SOP regarding roles and responsibilities are only present in 17%.

Italian current practice is to notify the physician with new arrhythmic episodes and questionable episodes (37%) or those not yet actioned (21%), while in a minority of cases all transmissions with episodes are notified (13%).

Our survey also shows high nurse confidence in reviewing remote data and notifying relevant information to the physician (97%) and high satisfaction (72%) about training materials on remote monitoring. Advancement in technology can facilitate nurse work, as demonstrated by 84% of surveyed nurses who overwhelmingly believe that tablet programmers make programming easier and faster and patient enrollment in the remote monitoring system more efficient compared to manual procedures. Nurses appreciate the dedicated technical support from manufacturers during remote patient management, while transmission screening by an external service [41] to the hospital and to the ICM manufacturer would be accepted only by 43% of nurses managing remote data review. AI holds promise for easing nurse workload because it reduces the number of transmitted false alerts that should be reviewed [42, 43, 44]. Though a significant portion (30%) had no opinion on AI effectiveness due to limited experience, 66% of nurses believe AI could reduce review times and 55% already trust the results of AI algorithms.

### 4.3 Limitations

The main limitations of our project stem from the voluntary nature of the survey. First, despite sending invitations to participate three times, we did not achieve a 100% response rate. Second, our sample may be subject to selection bias due to the predominance of public hospitals and limited representation from central Italy. However, our primary aim was to assess the rate of nurses currently performing ICM implantation and remote follow‐up among those trained, which our sample reflects. In addition, with nearly 70% participation, our results are representative of current practices among trained nurses in Italy. Third, although we could not verify survey responses in the clinical setting, the anonymity and voluntary nature of participation should have encouraged honest responses. Further studies comparing characteristics, organizational models, applied standard protocols, complication rates, and patient satisfaction at sites with and without nurse‐led implantation are needed to validate SOP for nurse‐led ICM implantation programs in both national and international multicenter settings.

## 5 Conclusions

While many Italian hospitals are motivated to train nurses in ICM implantation and remote management, only 60% allow trained nurses to perform implantations, and 71% can do so independently, without physician support. Over 70% of nurses are involved in remote patient management. The lack of regulations and SOP poses significant barriers to broader delegation of these responsibilities to nurses. Establishing clear guidelines at both national and international levels could standardize training and enhance awareness of effective practices, ultimately improving patient care in ICM management.

## Ethics Statement

The survey was fully anonymous, and no sensitive data were collected or managed. Participation of survey recipients was voluntary. The project was conducted in accordance with local laws and regulations.

## Consent

The authors have nothing to report.

## Conflicts of Interest

Rosario Cervellione is a proctor for Medtronic, and Massimiliano Maines is a consultant and proctor for Medtronic, Boston Scientific, and Abbott. The other authors declare no conflicts of interest.

## Data Availability

The data set generated and analyzed in this project is not publicly available but is available from the corresponding author at a reasonable request.

## Appendix A

English Translation of the Nurse Survey.

**Table:**

**Information about hospitals and available protocols related to loop recorder (ILR) implantation by nurse**	
1. Geographical area	North Center South and Island
2. Type of hospital	Public University public Affiliated private University Affiliated private
3. What is the number of loop recorders (ILR) implants/year at your hospital	Unknown < 10 10–20 20–50 > 50
4. In your hospital, which brands of ILR are used? (*report all applicable answers*)	Medtronic Boston Biotronik Abbott
5. In your hospital, what percentage of ILR implants are performed by nurses?	The nurse does not implant the ILR < 30% 30%–60% 60%–90% > 90%
*Only if in the hospital nurses do not implant ILR*, 6. What are the reasons? (*report all applicable answers*)	The physicians or nursing coordinator do not allow nurses to perform this procedure Some nurses do not want to take on such responsibility There is no a regulatory path available for this within the hospital The nursing staff has not yet completed the training pathway required by the hospital Despite proposal by nurse to perform ICM implantations, it was not possible to discuss the matter with hospital director The topic has not yet been seriously addressed Hospital administration did not authorize it Other (specify): …………………………
7. In your hospital, nurses perform ILR explantant procedures?	Yes No
8. In the case of loop recorder implantation by the nurse, how many additional operators are involved in the implantation procedure (excluding the supervisor during the training phase)?	Implanter nurse is alone 1 allied healthcare professional (nurse/healthcare assistants or cardiology technicians) 1 physician 1 physician + 1 allied healthcare professional (nurse/healthcare assistants or cardiology technicians) 2 allied healthcare professionals (nurse/healthcare assistants or cardiology technicians) If allied healthcare professionals are involved please specify: (nurse/healthcare assistants or cardiology technicians etc.): ________________________
9. In your hospital, there is a written procedure/protocol issued by the hospital director about ICM implantation by nurses?	Yes No
10. In your hospital, is it defined the minimum number of implants that must be performed by the nurse with a supervisor before being autonomous?	Yes No, the supervisor decides when nurse autonomy is achieved
*If yes to the previous* 11. What is the minimum number of implant procedures with a supervisor?	3 5 10 Between 10 and 20 ≥ 20
12. In your hospital is there a written documentation of ICM performed by nurses under supervision with date of achieved autonomy?	Yes No
13. In your hospital, is it necessary that nurses are periodically reassessed by a supervisor to continue performing ILR implantation?	Yes No
14. In your hospital, is a written tracking system of peri‐procedural complications (registry, database, form, etc.) available, including those related to ILR implantation?	Yes No
15. In your hospital, how are ILR complications monitored during follow‐up?	Education of patient about possible complications and ask him to report them by phone if they occur Schedule a visit after implantation to check wound Patient interview about ICM complication occurrence during in‐office or telephone follow‐ups
16. In your hospital, how many days after implant an in‐office follow‐up is carried out to check the wound?	In‐hospital follow‐up visit not scheduled 7 days Between 7 and 14 days ≥ 15 days
**Nurse experience**	
17. How many years of experience in cardiac pacing area do you have?	No experience in cardiac pacing area < 1 1–3 > 3
18. Do you perform ILR implantation at your hospital?	Yes No
If yes question 18	
19. Why did you choose to be part of the team of nurses who implant loop recorders? (*report all applicable answer*s)	It was an imposition Others do it and I do not want to be outdone I consider it professional growth I do it, but I would like to be recognized
20. How many ILR implants have you performed in total?	< 10 Between 10 and 20 ≥ 20
21. How many ILR implants have you performed under supervision, before becoming independent?	< 5 Between 5 and 10 Between 10 and 20 ≥ 20
22. It has never happened that the patient was against the nurse performing the implant.	Yes No
**Current nurse practice on ICM implant settings, tools, and implantation procedure** (*only if Yest at question 18*)	
23. In your hospital, where do you perform ICM implantation procedure and in what proportion? (*report all applicable answers and indicative percentages for each*)	EP/Cath lab or operating room: …….% Recovery room: …….% Dedicated in‐hospital out‐patient clinic with infection control (certified by hospital): …….% Non‐dedicated in‐hospital out‐patient clinic…….% Patient's bed in the ward: …….%
24. Where is the doctor mostly located when you are performing ILR implantation? (*report all applicable answers*)	Same room Nearby room On the same floor In another floor
25. When ICM implantation is not performed in the EP/Cath lab or operating room, what is the room/clinic equipped with for the procedure? (*report all applicable answers*)	Unknown, no implant performed in out of EP/Cath/OR settings Sink Multi‐parameter monitor Oxygen line Defibrillator Resuscitation trolley Telephone
26. If not present in the implant room/clinic, is there a resuscitation trolley available near the room/clinic where the implant is performed?	The resuscitation trolley is present in the implant room/clinic The resuscitation cart is nearby There is no resuscitation cart nearby
27. What do you wear for ICM implantation in out of EP/Cath/OR settings? (*report all applicable answers*)	Cap Surgical mask Aseptic gown A pair of sterile gloves 2 pairs of sterile gloves protective glasses
28. In patients on DOA or Coumadin, is this therapy temporarily stopped before ICM implantation? If yes, how long before?	No Yes, 12 h before ICM implantation Yes, 24 h before ICM implantation Yes, 48 h before ICM implantation
29. Which tools do you have during ICM implants? (*report all applicable answers*)	Sterile drapes with holes for the patient Backhaus Disinfectant Local anesthetic Syringe Sterile gauzes Scissors Anatomical forceps Needle holder Scalpel Absorbable sutures Nonabsorbable sutures Plasters Steri‐strip Skin glue Advanced dressings
30. During your ILR implantation, is patient EKG monitoring through the multiparameter monitor performed?	Yes No
31. During your ILR implantation, is patient venous access created?	Yes No
32. Which local anesthetic drugs do you use for ILR implantation?	Lidocaine Mepivacaine Ropivacaine Other (specify): ………….
33. Do you administer peri‐procedural antibiotic therapy for ILR implantation?	No Yes, in all patients Yes, in patients with high infection risk
34. During the implantation phase, do you use only the kit supplied by the manufacturing company?	Yes, Only the injection kit No, Injection kit + additional scalpel No, Injection kit + other (specify): ___________
35. Do you follow ILR implantation instructions defined by the manufacturer about position in the patient's chest, inclination of the device, etc.?	Yes, if patient's anatomy allows it Yes, if patient anatomy allows it, but with exception of women with breast implants No, pre‐implant EKG mapping is performed No, decision per implanter discretion
36. How do you most often close the wound? (*report all applicable answers*)	Steri‐Strips Absorbable stitches Nonabsorbable stitches Skin glue Nothing
37. What type of dressing do you apply? (*report all applicable answers*)	Medicated transdermal patches Medicated transdermal patches + application of ice and weight Advanced dressings (e.g., silver‐impregnated dressings) Compressive dressings Common gauze and plaster
38. Do you take care of ILR programming, according to physician indications? If not, who does it?	Yes, alone Yes, together with a cardiology technician No, another nurse manages it No, a cardiology technician manages it No, a physician manages it
**Nurse feedback and satisfaction with ICM implantation**	
How much do you agree with the following statements? (*select only one answer for each statement*)	
39. The insertion tool provided by the company makes the procedure easy, fast, and safe	I do not know I totally agree I agree I neither agree nor disagree I disagree I totally disagree
40. Once the training course (theoretical and practical) for the implant has been completed, I am able to carry out the implant procedures safely	I do not know I totally agree I agree I neither agree nor disagree I disagree I totally disagree
41. I find it rewarding to carry out the loop implantation procedure	I do not know I totally agree I agree I neither agree nor disagree I disagree I totally disagree
42. Manage loop implantation procedures increased my workload too much	I do not know I totally agree I agree I neither agree nor disagree I disagree I totally disagree
43. I'm not paid enough to even take care of the loop recorder implant procedure	I do not know I totally agree I agree I neither agree nor disagree I disagree I totally disagree
44. I rarely encounter a complication during the implant that requires the intervention of a physician (e.g., vagal episode, allergic reaction to anesthesia, and significant bleeding)	I do not know I totally agree I agree I neither agree nor disagree I disagree I totally disagree
45. In case of complications, I know how to promptly request physician's attention	I do not know I totally agree I agree I neither agree nor disagree I disagree I totally disagree
46. I am sure that in case of complications, the physician will intervene promptly	I do not know I totally agree I agree I neither agree nor disagree I disagree I totally disagree
(Only if Yes at question 38) 47. I believe that using the tablet makes programming quicker and easier than the traditional programmer	I do not know I totally agree I agree I neither agree nor disagree I disagree I totally disagree
**Information about ICM remote monitoring in the hospital, available protocols, and involvement of nurses**	
48. How many patients with loop recorders do you manage with the remote monitoring system?	< 50 50–200 200–500 > 500
49. What percentage of patients with ILR in your center are equipped with a remote monitoring system?	> 95% 75%–95% 50%–75% 25%–50% < 25%
50. In your hospital, when the patient is enrolled in the remote monitoring system?	At implant After implant, but pre‐discharge
51. In your hospital, does patient informed consent inform about the possibility of having remote monitoring withdrawn at a later date?	Yes No
52. In your hospital, do you enroll the patient in the remote monitoring system? If not, who does it?	Yes No, another nurse manages it No, a cardiology technician manages it No, a physician manages it
53. In your hospital, do you manage patient training about remote monitoring and symptom recording?	Yes No, another nurse manages it No, a cardiology technician manages it No, a physician manages it No, an external service provided by ICM manufacturer manages it
54. In your hospital, do you perform first review of remote data transmissions and notify the physician of the relevant ones?	Yes No, another nurse manages it No, a cardiology technician manages it No, a physician manages it
55. In your hospital, does a written protocol, or document of the hospital indicating which transmissions/events need to be notified to the physician, exist?	Yes No, but nurse and physician agree on which transmissions/events need to be notified No, a physician reviews ICM transmissions Unknown
56. In your hospital which type of transmissions are notified by nurses to the physicians?	All transmissions containing arrhythmic episodes Transmissions containing new and dubious arrhythmic episodes Transmissions containing new and non‐managed arrhythmic episodes I do not know; I am not involved in transmission review
57. In your hospital, what percentage of patients with ILR are provided with the possibility of activating the recording during symptoms?	> 95% 75%–95% 50%–75% 25%–50% < 25%
**Nurse feedback about enrollment in the remote monitoring system, training materials for nurses and patients, and ICM data transmission review**	
How much do you agree with the following statements? (*select only one answer for each statement*)	
(only if Yes at question 52) 58. I believe that the tablet is an added value for me and my colleagues who manage patient enrollment in the remote monitoring system and it is more efficient than manual enrollment on the website	I do not know I totally agree I agree I neither agree nor disagree I disagree I totally disagree
(only if Yes at question 53) 59. I am satisfied with training materials for nurses about remote monitoring	I do not know I totally agree I agree I neither agree nor disagree I disagree I totally disagree
(only if Yes at question 53) 60. I am satisfied with training materials for the patient about remote monitoring	I do not know I totally agree I agree I neither agree nor disagree I disagree I totally disagree
If yes, at question 54	
61. I believe that after adequate training, I will be able to notify the physician of relevant transmissions	I do not know I totally agree I agree I neither agree nor disagree I disagree I totally disagree
62. Where available, I find a dedicated support service from ICM manufacturing company useful during remote patient management	I do not know I totally agree I agree I neither agree nor disagree I disagree I totally disagree
63. When available, I believe AI reduces the time required to review ICM data transmissions	I do not know I totally agree I agree I neither agree nor disagree I disagree I totally disagree
64. I trust AI algorithms	I do not know I totally agree I agree I neither agree nor disagree I disagree I totally disagree
65. I would agree that a service external to the hospital and ICM manufacturing company would screen the transmissions and send me only those on which to decide whether to intervene	I do not know I totally agree I agree I neither agree nor disagree I disagree I totally disagree, Specify why: ………………………………………………….
